# Single nucleotide polymorphisms of cytokine-related genes and association with clinical outcome in a Chagas disease case-control study from Brazil

**DOI:** 10.1590/0074-02760170489

**Published:** 2018-05-14

**Authors:** Lucia Elena Alvarado-Arnez, Angelica Martins Batista, Silvia Marinho Alves, Gloria Melo, Virgínia Maria Barros de Lorena, Cynthia C Cardoso, Isabela Resende Pereira, Cristina Carrazzone, Antonio G Pacheco, Wilson Oliveira, Milton Ozório Moraes, Joseli Lannes-Vieira

**Affiliations:** 1 Fundação Oswaldo Cruz-Fiocruz, Instituto Oswaldo Cruz, Laboratório de Biologia das Interações, Rio de Janeiro, RJ, Brasil; 2 Ambulatório de Doença de Chagas e Insuficiência Cardíaca do Pronto Socorro Cardiológico de Pernambuco, Recife, PE, Brasil; 3 Fundação Oswaldo Cruz-Fiocruz, Instituto Aggeu Magalhães, Departamento de Imunologia, Laboratório de Imunoparasitologia, Recife, PE, Brasil; 4 Universidade Federal do Rio de Janeiro, Instituto de Biologia, Departamento de Genética, Laboratório de Virologia Molecular, Rio de Janeiro, RJ, Brasil; 5 Fundação Oswaldo Cruz-Fiocruz, Programa de Computação Científica, Rio de Janeiro, RJ, Brasil; 6 Fundação Oswaldo Cruz-Fiocruz, Instituto Oswaldo Cruz, Laboratório de Hanseníase, Rio de Janeiro, RJ, Brasil

**Keywords:** Chagas disease, Trypanosoma cruzi, cardiomyopathy, cytokines, gene polymorphism, meta-analysis

## Abstract

**BACKGROUND:**

The severity of chronic chagasic cardiomyopathy (CCC), the most frequent clinical outcome of Chagas disease (CD), has been associated with cytokine-enriched heart tissue inflammation, and high serum levels of transforming growth factor (TGFβ), interferon-gamma (IFNγ), and tumour necrosis factor (TNF). Conversely, increased interleukin (IL)-10 serum concentrations have been associated with asymptomatic CD. Cytokines and cytokine-related gene polymorphisms may control cytokine expression and have been proposed to contribute to CCC outcomes.

**OBJECTIVES:**

We evaluated the association of 13 cytokine-related genes (***TGFB***: rs8179181, rs8105161, rs1800469; ***IL10***: rs1800890, rs1800871, rs1800896; ***IFNG***: rs2430561; ***TNF***: rs1800629; ***BAT1***: rs3853601; ***LTA***: rs909253, rs2239704; ***TNFR1***: rs767455; ***TNFR2***: rs1061624) with risk and progression of CCC.

**FINDINGS:**

Four hundred and six seropositive patients from CD endemic areas in the state of Pernambuco, north-eastern Brazil, were classified as non-cardiopathic (A, 110) or cardiopathic (mild, B1, 163; severe, C, 133). We found no evidence of *TGFB*, *IL10*, *TNF*, or *TNFR1/2* gene polymorphisms associated with CCC risk or progression. Only *BAT1* rs3853601 −22G carriers (B1 *vs.* C: OR = 0.5; p-value = 0.03) and *IFNG* rs2430561 +874AT (A *vs.* C: OR = 0.7; p-value = 0.03; A *vs.* B1+C: OR = 0.8; p-value = 0.02) showed a significant association with protection from cardiopathy in a logistic regression analysis with adjustment for gender and ethnicity; however, the association disappeared after performing adjustment for multiple testing. A systematic review of *TNF* rs1800629 −308G>A publications included five studies for meta-analysis (534 CCC and 472 asymptomatic patients) and showed no consensus in pooled odds ratio (OR) estimates for A allele or A carriers (OR = 1.4 and 1.5; p-values = 0.14 and 0.15, respectively). In CD patients, TNF serum levels were increased, but not affected by the *TNF* rs1800629 −308A allele.

**MAIN CONCLUSIONS:**

Our data suggest no significant contribution of the analysed gene variants of cytokine-related molecules to development/severity of Chagas' heart disease, reinforcing the idea that parasite/host interplay is critical to CD outcomes.

Chagas disease (CD), a neglected disease caused by the protozoan parasite *Trypanosoma cruzi*, afflicts ~10 million in Latin America (Herricks et al. 2017). In the last decades, due to immigration, *T. cruzi*-infected individuals have raised the attention of people working in the healthcare systems of North America and Europe (Herricks et al. 2017). Acute infection is mostly asymptomatic, and 10-30 years after infection 60-70% of infected individuals show no clinical signs, remaining in the indeterminate form of CD. Nevertheless, 20-30% of these CD patients developed the cardiac form of CD, chronic chagasic cardiomyopathy (CCC), which shows a spectrum of severity from mild to severe, with heart tissue inflammation and remodelling, fibrosis, electrical and structural abnormalities, and thromboembolic events, culminating in heart failure (Rassi Jr et al. 2017). The pathogenic determinants of this range of clinical manifestations remain unclear. Genetic factors may determine the form of the disease and severity of the cardiac form (Williams-Blangero et al. 2011). Several studies have implicated parasite load and/or diversity, as well as the systemic inflammatory profile with enrichment in cytokines and inflammatory mediators, as determinants of the severity of CD (Cunha-Neto and Chevillard 2014, Rassi Jr et al. 2017). As a theoretical framework, we assumed that clinical manifestation and severity of Chagas' heart disease are multifactorial processes related to (i) parasite persistence and (ii) an inappropriate or dysregulated host immune response, which may result from genetic predisposition and/or interplay between the immune system and the parasite. In the present study, we will focus on the role of cytokines and cytokine-related gene polymorphisms as contributing factors for the development and severity of Chagas' heart disease.

In CD patients, serum concentrations of transforming growth factor (TGF)-β have been associated with heart fibrosis intensity, disease progression, and mortality (Saraiva et al. 2013). Several studies have revealed that high tumour necrosis factor (TNF) levels in serum are associated with the development and severity of the cardiac form of CD (Sousa et al. 2014). High IL-10 expression has been observed in patients with the indeterminate form of CD, but not in patients with the cardiac form (Sousa et al. 2014), supporting a lack of immunoregulatory machinery in CCC patients.

Cytokines play crucial roles in the regulation of immune responses. Gene polymorphisms may determine differential cytokine expression, which can contribute to clinical features, ranging from the development of immunological disorders to susceptibility to infections or disease severity. Several variants in different genes have been studied in *T. cruzi*-infected South American populations, mostly Brazilians, Colombians, and Peruvians. The most commonly studied single nucleotide polymorphisms (SNPs) are *TNF*, *LTA*, *BAT1*, *TGFB*, and *IL10*, and, although some associations with risk or progression of Chagas' heart disease have been described, there is a need to replicate the findings in different populations (Beraún et al. 1998, Rodríguez-Pérez et al. 2005, Ramasawmy et al. 2006, Drigo et al. 2007, Calzada et al. 2009, Costa et al. 2009, Flórez et al. 2011, Pissetti et al. 2013).

In CD, association studies of cytokine gene polymorphisms are restricted in number, frequently have a small number of patients, and, mostly, are not conclusive regarding the development/progression of the cardiac form of disease as an outcome. The present study was designed to determine whether SNPs in key molecules in the immune response to *T. cruzi* infection and immunoregulation (Cunha-Neto and Chevillard 2014), namely *TGFB* (rs8179181, rs8105161, rs1800469), *IL10* (rs1800890, rs1800871, rs1800896), interferon-gamma (*IFNG*) (rs2430561), *TNF* (rs1800629), and its receptors *TNFR*1/2 (*TNFR1*: rs767455; *TNFR2*: rs1061624), *LTA* (rs909253, rs2239704), and *BAT1* (rs3853601), are associated with the development and progression of CCC. We focused our study on a group of *T. cruzi*-seropositive patients from the northeast region of Brazil that have not been evaluated in previous studies for CD and genetic polymorphisms. Patients from the state of Pernambuco were classified in cardiomyopathy stages accordingly to the I Latin American Guideline for the Diagnosis and Treatment of Chagas' Heart Disease (Andrade et al. 2011). Further, we summa-rise previously reported studies for the *TNF* rs1800629 −308G>A polymorphism to obtain a consensus association estimation by means of meta-analysis.

## MATERIALS AND METHODS

*Study subjects* - A group of 406 patients that attended to the Ambulatório de Doença de Chagas e Insuficiência Cardíaca do Pronto Socorro Cardiológico de Pernambuco (PROCAPE)/Universidade do Estado de Pernambuco (UPE) were enrolled (July 2010 - June 2015) in an unmatched case-control study. The recruited patients had serological diagnoses of CD based on at least two positive tests: enzyme-linked immunosorbent assay (ELISA), western blotting, and/or indirect immunofluorescence, performed by the Central Reference Laboratory (LACEN) of Pernambuco, Brazil, in accordance with the 1st (2005) and 2nd (2015) Brazilian Consensus on Chagas Disease (Dias et al. 2016). At baseline, the patients were evaluated by anamnesis, and 12-lead electrocardiography findings were recorded (ECG; Ecafix, São Paulo, SP, Brazil). Two-dimensional and M-mode echocardiography (ECHO) doppler was performed using a Vivid 3 (GE Health Care, Wauwatosa, WI, USA), and digital images were recorded. Participants were classified into three (A, B1, and C) different clinical groups accordingly to the I Latin American Guideline for the Diagnosis and Treatment of Chagas' Heart Disease (Andrade et al. 2011). Patients under 18 years of age or with digestive or cardio-digestive forms of CD and co-infections were excluded. The study, with the number of patients included in each clinical group, had 76% statistical power to detect a genetic association of 1.85, estimated with the lower minor allele frequency of 0.10 under the additive model.

*DNA extraction and SNP genotyping* - Genomic DNA was isolated from frozen blood samples using a precipitation salting out technique. After extraction, DNA was quantified using a NanoDrop ND-1000 Spectrophotometer (NanoDrop Technologies, USA). Altogether, we analysed 13 SNPs using TaqMan assays [Supplementary data (Table I)]. Reactions were performed with 30 ng of yield DNA for each sample following the manufacturer's recommendations for allelic discrimination using a ViiA 7 Real Time polymerase chain reaction (PCR) System (Applied Biosystems, USA). SNPs were analysed for the cluster *IL10-TGFB1* and *IFNG*: rs1800890, rs1800871, rs1800896, rs8179181, rs8105161, rs1800469, and rs2430561; the cluster *TNF-BAT-LTA*: rs1800629, rs3853601, rs909253, and rs2239704; and the cluster *TNFR1-TNFR2*: rs767455 and rs1061624. Raw genotyping data were deposited at https://www.arca.fiocruz.br/handle/icict/20995.

*Functional analysis* - Serum levels of TNF were quantified using ELISA DuoSet (R&D Biosystems, Minneapolis, MN) according to manufacturer's instructions. Data were analysed (GraphPad Prism software version 5.0 for Windows) by comparing the levels of cytokines: (i) correlating with the left ventricular ejection fraction (LVEF; %); (ii) between the different clinical groups; and (iii) stratifying cytokine levels according to *TNF*-308 SNP genotypes. For this study, 10 triatomineexposed and region-matched individuals excluded from our genetic study because of negative *T. cruzi* serology were included as non-infected controls.

*Statistical analysis* - Analyses were performed in R environment version 3.3.3 using the following packages: coin, epiDisplay, gap, genetics, stats, haplo.stats, meta, metafor, and SNPassoc. The comparison amongst demographic or clinical variables was performed either by Chi-square or Kruskal-Wallis test when appropriate, and the frequencies of each variable excluded missing data. Genotypic and minor allele carrier frequencies were determined, and associations with CD were evaluated by means of unconditional logistic regression comparing individuals at different stages of the cardiac form of CD disease. Two non-genetic variables, gender and ethnicity, were included as categorical covariates in the regression model. First, we tried to determine the influence of SNPs on developing the cardiac form of CD. For that, patients from group A were considered controls, and patients from B1 and C groups were considered cases. Then, to examine the influence of SNPs on severity of CD cardiomyopathy, people in group B1 (mild CCC) were considered controls, and people in group C (severe CCC) were considered cases. Allele-dose effects were tested with the Cochran-Armitage trend test. Also, the false discovery rate (FDR) was determined to adjust for multiple testing in the different genetic models. For polymorphisms located within the same gene, haplotype frequencies were estimated by the maximum likelihood method and compared using the same unconditional regression models as used for the analysis for individual SNPs. A linkage disequilibrium (LD) analysis, evaluated trough r^2^ statistics, was performed using Haploview software separately for *TGFB1, IL10*, and *TNF-BAT-LTA* clusters. Finally, the analysis of TNF serum levels was performed using GraphPad Instat software version 3.0 (San Diego, CA). Statistical significance was considered with p*-*values < 0.05, with a Bonferroni correction applied when ANOVA tests were performed.

*Meta-analysis* - Published articles that evaluated the association between CD and gene polymorphisms were identified in databases such as MEDLINE using PubMed (http://www.ncbi.nlm.nih.gov/pubmed) and the Cochrane library (http://www.cochranelibrary.com/). The search keywords were a combination of: “cytokines” and “Chagas disease”, “polymorphism(s)” and “SNP(s)”. In addition, when evaluating each article individually, we reviewed reference lists and related citations suggested by PubMed to broaden our results. We did not use SNP rs identification numbers in the search. As inclusion criteria, we considered studies if they were published up to March 2017 and provided sufficient genotypic data to determine allelic counts for the analysis. Studies were excluded if they were related to a previous publication or if the control individuals deviated from Hardy-Weinberg equilibrium (HWE).

**Fig. 1:**
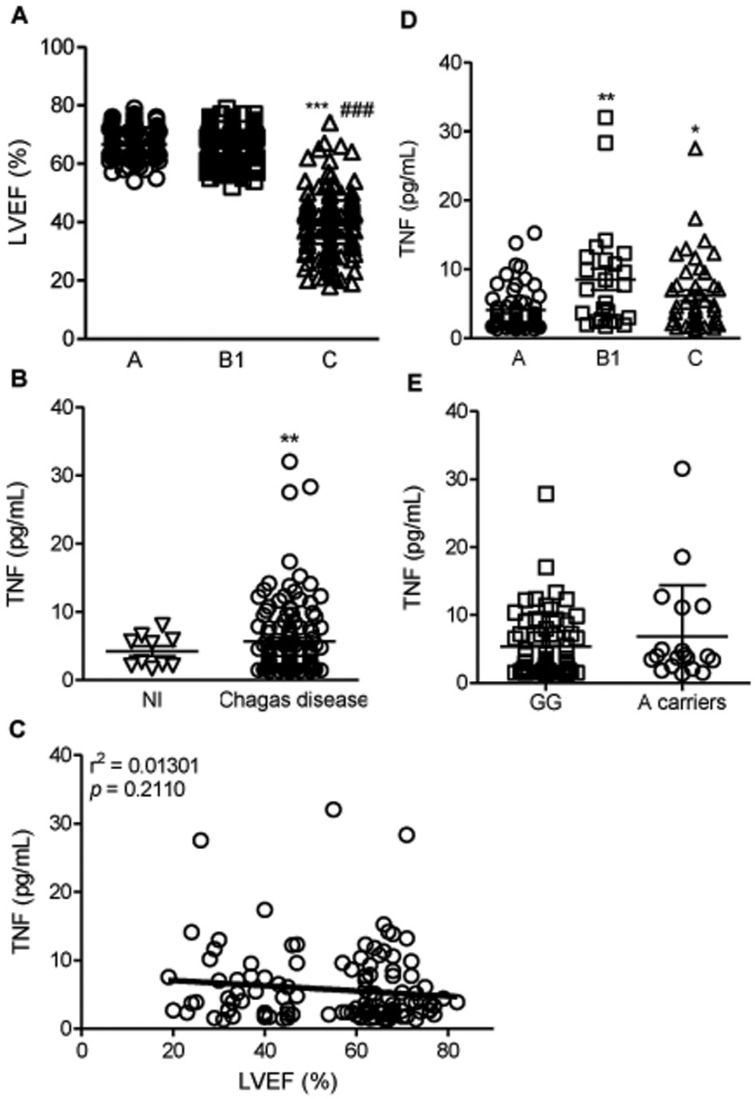
TNF levels and association with left ventricular ejection fraction (LVEF) and genotypes in *Trypanosoma cruzi*-infected patients. (A) LVEF of CD patients grouped as A (asymptomatic), B1 (mild CCC), and C (severe CCC); ^***^, p < 0.001 group C *vs.* A; ^###^, p < 0.001 group C *vs.* B1 (Kruskal-Wallis test). (B) TNF levels in the sera of subjects who were seronegative and considered non-infected (NI) and those that were seropositive for Chagas disease; ^**^, p < 0.01 (*t*-student test). (C) Correlation between TNF serum concentrations and LVEF in patients; p = 0.2110, r^2^ = 0.01301 (linear regression). (D) TNF concentrations in the sera of CD patients grouped as A (asymptomatic), B1 (mild CCC), and C (severe CCC); ^*^, p < 0.05, ^**^, p < 0.01 group C and B1 *vs.* A (ANOVA, Bonferroni post-test). (E) Comparison of subjects according to the presence or absence of allele A (Mann-Whitney test).

Two authors (LEAA and AMB) independently extracted genotype counts from the studies that met the inclusion criteria [Supplementary data (Fig. 1)]. The recorded information included variables such as first author, year in which the study was published, population that was evaluated, age and number of females and males for both cases and controls, source of control individuals, genotyping method, and, finally, genotype counts for cases and controls.

We used a Chi-square test to determine whether genotype frequencies in the control groups of each of the selected studies followed HWE. Publication bias was evaluated by Egger's test to provide statistical evidence for funnel plot symmetry. Heterogeneity across studies was established by Cochran's Q statistic. Pooled OR estimates were obtained by DerSimonian and Laird random effects model referencing to the minor allele for each polymorphism. Forest plots represented individual OR values for each study and pooled ORs also referring to the minor allele for the studied SNPs. To determine the effect of each study on the overall OR, we performed a sensitivity analyses in which individual studies were removed sequentially. Adjustment for environmental effects and population stratification were not performed due to the lack of such covariates. The statistical calculations were carried out in *R.*

*Ethics* - Signed informed consents were obtained from all patients included in the study. All study protocols were approved by the Ethics Committees of Fiocruz/RJ (541/09) and PROCAPE/UPE (80210/10).

## RESULTS

*Epidemiological data and clinical classification of the enrolled patients* - The main characteristics of the enrolled individuals are shown in Table I. The recruited individuals were classified as stage A, a group of 110 patients without cardiac symptoms and with normal ECG and ECHO; stage B1, a group of 163 patients with structural cardiopathy, evidenced by ECG or ECHO changes, but with normal global ventricular function and neither current nor previous signs and symptoms of congestive heart failure (CHF); and stage C, a group of 133 patients with ventricular dysfunction and current or previous symptoms of CHF. Mean age and standard deviation for each of the clinical groups were as follows: A (51 ± 12), B1 (60 ± 13), and C (60 ± 11), although more than 65% of patients in either group were in the > 45-year-old stratum. The distribution of patients according to gender showed a high frequency of females in groups A (64.5%), B1 (77.3%), and C (63.9%). Regarding ethnicity, most of the patients identified themselves as mestizo. Independent of the clinical stage, most of the patients had a monthly income of up to one minimum wage (MW) and an education level of up to four years. As an important clinical variable, the LVEF was similar in patients of group A (67% ± 5%) and group B1 (66% ± 6%), but showed a significant decrease in patients of group C (40% ± 11%, p-value < 0.001), as shown in Table I and Fig. 1A. The use of the trypanocidal drug benznidazole was registered in 44.5% of the patients in stage A, 11.6% in stage B1, and 14.3% in stage C (Table I).

**TABLE I:** Demographic, epidemiological and clinical data of the recruited patients

Variable^a^		A Group N = 110	B1 Group N = 163	C Group N = 133	p-value
Gender					
	Female	71 (64.5)	126 (77.3)	85 (63.9)	
	Male	39 (35.5)	37 (22.7)	48 (36.1)	
Age (years)^b^		51 ± 12	60 ± 13	60 ± 11	KW < 0.001
	≤ 45	37 (33.6)	23 (14.4)	17 (12.8)	
	> 45	73 (66.4)	138 (85.7)	116 (87.2)	
Ethnicity (n = 353)		94	141	118	Chisq. 0.34
	White	27 (28.7)	26 (18.4)	27 (22.9)	
	Black	8 (8.5)	11 (7.8)	13 (11.0)	
	Mestizo	59 (62.8)	104 (73.8)	78 (66.1)	
Monthly income (n = 377)		106	14 9	122	Chisq. 0.82
	Up to 1 MW	70 (66.7)	99 (66.5)	88 (72.1)	
	2-4	14 (13.3)	23 (15.4)	14 (11.5)	
	More than 5	21 (20.0)	27 (18.1)	20 (16.4)	
Education (n = 379)		103	154	122	Chisq. 0.14
	Up to 4 years	84 (81.6)	123 (79.9)	108 (88.5)	
	More than 4 years	19 (18.4)	31 (20.1)	14 (11.5)	
Region (n = 358)		101	153	121	Chisq < 0.001
	PE, Agreste	25 (24.8)	22 (14.4)	31 (25.6)	
	PE, Mata	21 (20.8)	79 (51.6)	52 (43.0)	
	PE, Metropolitan	6 (5.9)	8 (5.2)	6 (5.0)	
	PE, Sertão	27 (26.7)	31 (20.3)	14 (11.6)	
	Other States	22 (21.8)	13 (8.5)	18 (14.9)	
	Clinical information	110	16 3	133	
LVEF (%)^b^		67 ± 5	66 ± 6	40 ± 11	KW < 0.001
	Benznidazole	49 (44.5)	19 (11.6)	19 (14.3)	

a results are presented as number (frequency);

b mean ± standard deviation; n: number of patients; MW: minimum wage; PE: state of Pernambuco; LVFE: left ventricular ejection fraction.

*Polymorphisms at TGFB, IFNG, IL-10, TNF, LTA, and BAT1 genes and association with Chagas' heart disease* - A call rate efficiency of 90% was obtained for all polymorphisms studied. The study of the rs8179181 (+20743 C>T), rs8105161 (+19318 A>G) and rs1800469 (-509 C>T) polymorphisms at *TGFB* gene showed no significant association when comparing susceptibility to or severity of CCC (Table II). Similarly, the *IL10* SNPs rs1800890 (-3575 T>A), rs1800896 (-1082 G>A), and rs1800871 (-819 C>T) did not act as a genetic modifier of Chagas' heart disease outcome (Table III). Interestingly, the *IFNG* SNP rs2430561 (+874 T>A) showed a significant association with protection to CCC development, when comparing non-cardiopathic (A) *vs.* mildcardiopathic (B1) patients that were genotype AT (OR = 0.7, *P*-value = 0.03) and A carriers (OR = 0.6, p-value = 0.06) and non-cardiopathic (A) *vs.* cardiopathic (B1+C) patients that were genotype AT (OR = 0.8, p-value = 0.02) and A carriers (OR = 0.6, p-value = 0.06), as described in Table III. Total genotype counts could vary because of differences in genotypic call rates.

**TABLE II:** Analysis of single nucleotide polymorphisms located in *TGFB1* gene

	Stage A N=110	Stage B1 N=163	Stage C N= 133	A versus B1	A versus C			B1 versus C		A versus B1+C	
				OR^a^ [95% CI]	p-value	OR^a^ [95% CI]	p-value	OR^a^ [95% CI]	p-value	OR^a^ [95% CI]	p-value
+20743 C > T rs8179181											
CC	68 (0.74)	101 (0.72)	81 (0.71)				Reference				
AC	23 (0.25)	36 (0.26)	31 (0.27)	1.1 [0.6-2.0]	0.86	1.1 [0.6-2.1]	0.78	1.1 [0.6-1.9]	0.95	1.1 [0.6-1.9]	0.75
AA	1 (0.01)	3 (0.02)	2 (0.02)	1.7 [0.2-174]		2.1 [0.2-24.8]		0.84 [0.1-5.4]		2.0 [0.2-18.2]	
A carriers	24 (0.26)	39 (0.28)	33 (0.29)	1.1 [0.6-2.1]	0.69	1.2 [0.6-2.2]	0.62	1.05 [0.6-1.8]	0.86	1.1 [0.6-2.0]	0.62
+19318A > G rs8105161											
GG	55 (0.60)	97 (0.70)	73 (0.63)				Reference				
AG	31 (0.34)	37 (0.27)	40 (0.35)	0.7 [0.4-1.2]	0.29	1.0 [0.5-1.8]	0.22	1.4 [0.8-2.4]	0.41	0.8 [0.5-1.4]	0.25
AA	6 (0.06)	5 (0.03)	2 (0.02)	0.5 [0.2-1.8]		0.3 [0.05-1.4]		0.6 [0.1-3.4]		0.4 [0.1-1.2]	
A carriers	37 (0.40)	42 (0.30)	42 (0.37)	0.6 [0.4-1.1]	0.12	0.9 [0.5-1.5]	0.61	1.3 [0.8-2.2]	0.34	0.7 [0.4-1.2]	0.25
-509 C > T rsl800469											
TT	32 (0.36)	54 (0.40)	41 (0.37)				Reference				
CT	47(0.53)	62 (0.47)	52 (0.48)	0.8 [0.2-1.1]	0.56	0.9 [0.5-1.6]	0.62	1.1 [0.6-2.0]	0.91	0.8 [0.5-1.4]	0.51
CC	10(0.11)	17(0.13)	16(0.15)	1.3 [0.8-1.8]		1.3 [0.5-3.3]		1.1 [0.5-2.5]		1.2 [0.5-2.8]	
C carriers	57 (0.64)	79 (0.60)	68 (0.62)	0.8 [0.2-1.5]	0.51	0.9 [0.5-1.7]	0.83	1.1 [0.7-1.9]	0.67	0.9 [0.5-1.4]	0.60

a odds ratio (OR) values shown are corrected for gender and ethnicity; results are shown as n (frequency); CI: confidence interval; total genotype counts can vary due to different genotypic call rates.

**TABLE III:** Analysis of single nucleotide polymorphisms located in *IL10* and *IFNG* genes

	Stage A N= 110	Stage B1 N= 163	Stage C N= 133	A versus B1	A versus C			B1 versus C		A versus B1+C	
				OR^a^ [95% CI]	p-value	OR^a^ [95% CI]	p-value	OR^a^ [95% CI]	p-value	OR^a^ [95% CI]	p-value
***IL10*-3575T > A rs1800890**											
AA	45 (0.59)	78 (0.57)	65 (0.58)				Reference				
AT	23 (0.31)	54 (0.39)	39 (0.35)	1.4 [0.8-2.7]	0.14	1.2 [0.6-2.2]	0.64	0.8 [0.5-1.4]	0.47	1.3 [0.7-2.3]	0.27
TT	8 (0.10)	5 (0.04)	8 (0.07)	0.4 [0.1-1.5]		0.7 [0.2-2.0]		1.6 [0.5-5.4]		0.5 [0.2-1.5]	
T carriers	31 (0.41)	59 (0.43)	47 (0.42)	1.2 [0.6-2.2]	0.57	1.0 [0.6-1.9]	0.86	0.9 [0.5-1.5]	0.66	1.1 [0.6-1.9]	0.66
***IL10*-1082G > A rs1800896**											
GG	51 (0.55)	61 (0.44)	56 (0.50)				Reference				
GA	31 (0.34)	68 (0.50)	47 (0.41)	1.9 [1.1-3.3]	0.07	1.4 [0.8-2.5]	0.50	0.7 [0.4-1.3]	0.48	1.6 [0.9-2.7]	0.15
AA	10 (0.11)	9 (0.06)	10 (0.09)	0.9 [0.3-2.4]		0.9 [0.4-2.4]		1.1 [0.4-3.0]		0.9 [0.4-2.1]	
A carriers	41 (0.45)	77 (0.56)	57 (0.50)	1.6 [1.0-2.8]	0.07	1.3 [0.7-2.2]	0.40	0.8 [0.5-1.3]	0.35	1.4 [0.9-2.3]	0.14
***IL10*-819C > T rs1800871**											
CC	25 (0.44)	57 (0.43)	38 (0.38)				Reference				
CT	26 (0.46)	60 (0.45)	48 (0.48)	1.0 [0.5-1.9]	0.97	1.2 [0.6-2.5]	0.63	1.2 [0.7-2.2]	0.74	1.1 [0.6-2.0]	0.78
TT	6 (0.10)	16 (0.12)	14 (0.14)	1.1 [0.4-3.2]		1.6 [0.6-4.9]		1.1 [0.5-2.7]		1.4 [0.5-3.8]	
T carriers	32 (0.56)	76 (0.57)	62 (0.62)	1.0 [0.5-1.9]	0.95	1.3 [0.7-2.6]	0.41	1.2 [0.7-2.1]	0.46	1.1 [0.6-2.1]	0.64
***IFNG*+874T > A rs2430561**											
TT	27(0.36)	72 (0.52)	53 (0.47)				Reference				
AT	28 (0.38)	53 (0.38)	44 (0.39)	0.7 [0.4-1.4]	**0.03**	0.8 [0.4-1.6]	0.10	1.2 [0.7-2.0]	0.77	0.8 [0.4-1.4]	**0.02**
AA	19 (0.26)	14 (0.10)	15 (0.13)	0.3 [0.1-0.7]		0.4 [0.2-0.9]		1.3 [0.6-3.0]		0.3 [0.2-0.7]	
A carriers	47 (0.64)	67 (0.48)	59 (0.52)	0.6 [0.3-1.0]	0.06	0.6 [0.4-1.2]	0.17	1.2 [0.7-2.0]	0.50	0.6 [0.3-1.0]	0.06

a odds ratio (OR) values shown are corrected for gender and ethnicity; results are shown as n (frequency); CI: confidence interval; rotal genotype counts can vary due to different genotypic call rates.

As shown in Table IV, out of the four studied SNPs harboured in the *TNF-BAT1-LTA* cluster, only *BAT1* rs3853601 −22 CG genotype and G allele carriers showed an association with protection (OR_CG_ = 0.8, p-value = 0.05 and OR_G_ = 0.5, p-value = 0.03) when analysing cardiomyopathy severity, comparing B1 patients as a control group and C patients as a case group. However, the significance of both associations (*IFNG* rs2430561 and *BAT1* rs3853601) was lost after performing adjustment for multiple testing (p*-*value > 0.05).

**TABLE IV:** Analysis of single nucleotide polymorphisms located in the *TNF-BAT-LTA* cluster

	Stage A N= 110	Stage B1 N= 163	Stage C N= 133	A versus B1	A versus C			B1 versus C		A versus B1+C	
				OR^a^ [95% CI]	p-value	OR^a^ [95% CI]	p-value	OR^a^ [95% CI]	p-value	OR^a^ [95% CI]	p-value
***TNF*-308 G > A rs1800629**											
GG	71 (0.76)	114 (0.82)	92 (0.80)				Reference				
GA	21 (0.22)	24 (0.17)	21 (0.18)	0.7 [0.4-1.4]	0.53	0.8 [0.4-1.5]	0.75	1.1 [0.6-2.1]	0.96	0.7 [0.4-1.3]	0.62
AA	1 (0.01)	1 (0.01)	1 (0.09)	0.6 [0.03-9.9]		0.8 [0.05-13.7]		1.2 [0.1-20.6]		0.7 [0.06-8.2]	
A carriers	22 (0.23)	25 (0.18)	22 (0.19)	0.7 [0.4-1.3]	0.26	0.8 [0.4-1.5]	0.45	1.1 [0.6-2.1]	0.79	0.7 [0.4-1.3]	0.33
***BAT1*-22 C** > **G rs3853601**											
CC	76 (0.84)	103 (0.76)	97 (0.86)				Reference				
CG	12 (0.13)	29 (0.21)	12(0.11)	2.0 [0.9-4.2]	0.18	0.8 [0.3-1.8]	0.79	0.4 [0.2-0.9]	0.05	1.3 [0.6-2.7]	0.71
GG	2 (0.02)	3 (0.03)	3 (0.03)	1.2 [0.2-7.5]		1.3 [0.2-7.9]		1.0 [0.2-5.4]		1.2 [0.2-6.3]	
G carriers	14 (0.15)	32 (0.23)	15 (0.13)	1.9 [0.9-3.8]	0.08	0.8 [0.4-1.8]	0.65	0.5 [0.2-0.9]	**0.03**	1.3 [0.7-2.5]	0.41
*LTA* **+252** A > G **rs909253**											
AA	36 (0.39)	56 (0.40)	53 (0.46)				Reference				
AG	44 (0.48)	67 (0.48)	49 (0.42)	1.0 [0.5-1.7]	0.93	0.7 [0.4-1.3]	0.54	0.8 [0.4-1.3]	0.62	0.8 [0.5-1.4]	0.80
GG	12 (0.13)	17 (0.12)	14 (0.12)	0.8 [0.4-2.0]		0.7 [0.3-1.8]		0.8 [0.4-1.9]		0.8 [0.4-1.8]	
G carriers	56 (0.61)	84 (0.60)	63 (0.54)	0.9 [0.5-1.6]	0.78	0.7 [0.4-1.3]	0.26	0.8 [0.5-1.3]	0.34	0.8 [0.5-1.4]	0.51
***LTA* +80 A** > **C rs2239704**											
CC	43 (0.46)	69 (0.50)	51 (0.44)				Reference				
AC	33 (0.35)	53 (0.38)	51 (0.44)	1.1 [0.6-1.9]	0.37	1.3 [0.7-2.4]	0.29	1.4 [0.8-2.3]	0.54	1.1 [0.7-1.9]	0.27
AA	17 (0.18)	16 (0.12)	14 (0.12)	0.6 [0.3-1.3]		0.7 [0.3-1.6]		1.2 [0.5-2.7]		0.6 [0.3-1.3]	
A carriers	50 (0.54)	69 (0.50)	65 (0.56)	0.9 [0.5-1.6]	0.72	1.1 [0.6-1.9]	0.72	1.3 [0.8-2.2]	0.29	0.9 [0.6-1.6]	0.90

a odds ratio (OR) values shown are corrected for gender and ethnicity; results are shown as n (frequency); CI: confidence interval; total genotype counts can vary due to different genotypic call rates.

To combine the SNPs located in the same gene, we performed a haplotype analysis for the *IL10* [Supplementary data (Table II)] and *TNF-BAT1-LTA* [Supplementary data (Table III)] clusters. The results revealed no association between *IL10* (rs1800890/rs1800896/rs1800871) and *TNF-BAT1-LTA* (rs1800629/rs3853601/rs909253/rs2239704) clusters with CCC outcome, as previously indicated for individual SNPs comparisons.

**Fig. 2:**
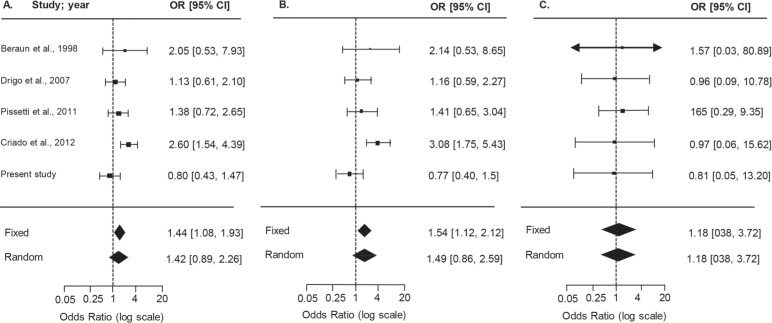
forest plots summarising the association between the *TNF* −308G>A SNP and susceptibility to Chagas' heart disease, based on allele, carrier, and genotype comparisons. Results from the random-effects model are shown. Bars represent 95% confidence intervals (CIs) and boxes represent the odds ratio (OR) values. The size of each box indicates the weight of the study in the pooled results.

A linkage disequilibrium analysis for each of the genetic clusters (*TGFB*, *IL10*, and *TNF-BAT-LTA*) and evaluating the CD patients recruited for the present study showed that, for the overall population of CD patients, r^2^ values were low (< 0.40), indicating no linkage dis-equilibrium among the markers in each of the clusters [Supplementary data (Fig. 2)].

*TNF levels in the serum and TNF −308 genotype association* - As expected, TNF levels in the serum were increased in *T. cruzi*-infected patients compared with those in non-infected controls putatively exposed to infection and born and residing in the same endemic areas (Fig. 1B). Although there was no correlation between TNF levels and reduction of LVEF (Fig. 1C), a significant increase in TNF levels in serum was detected in *T. cruzi*-infected patients of groups B1 and C, compared with asymptomatic patients in group A (Fig. 1D). Nevertheless, our data showed no association between the *TNF* rs1800629 −308 G>A genotype and TNF levels in serum of CD patients (Fig. 1E).

*Polymorphisms at TNFR1 and TNFR2 genes* - Considering that TNF signals via TNFR1 and TNFR2, we also analysed the possible influence of TNF receptors on CCC outcome. Gene variants of *TNFR1* (rs767455, +36 A>G) and *TNFR2* (rs1061624, +1466 A>G) showed no association with either development or progression of Chagas' heart disease in the studied groups (Table V).

**TABLE V:** Analysis of single nucleotide polymorphisms located in the *TNFR1* and *TNFR2* cluster

	Stage A N= 110	Stage B1 N= 163	Stage C N= 133	A versus B1	A versus C			B1 versus C		A versus B1+C		
				OR^a^ [95% CI]	p-value	OR^a^ [95% CI]	p-value	OR^a^ [95% CI]	p-value	OR^a^ [95% CI]	p-value
***TNFR1* +36 A > G rs767455**											
GG	47 (0.52)	66 (0.48)	46 (0.40)				Reference				
AG	36 (0.40)	58 (0.42)	56 (0.49)	1.1 [0.6-2.0]	0.86	1.6 [0.9-2.8]	0.24	1.3 [0.8-2.2]	0.58	1.3 [0.8-2.2]	0.44
AA	8 (0.08)	13 (0.10)	13(0.11)	1.2 [0.5-3.3]		1.8 [0.7-4.7]		1.3 [0.5-3.2]		1.5 [0.6-3.5]	
A carriers	44 (0.48)	71 (0.52)	69 (0.60)	1.2 [0.7-2.0]	0.60	1.6 [0.9-2.8]	0.09	1.3 [0.8-2.2]	0.30	1.4 [0.8-2.2]	0.21
***TNFR2*+1466 A > G rs1061624**											
AA	25 (0.27)	32 (0.23)	36 (0.32)				Reference				
AG	49 (0.53)	72 (0.53)	53 (0.46)	1.1 [0.6-2.2]	0.80	0.8 [0.4-1.5]	0.63	0.7 [0.4-1.2]	0.43	0.9 [0.5-1.6]	0.78
GG	18 (0.20)	33 (0.24)	25 (0.22)	1.3 [0.6-3.0]		1.0 [0.4-2.2]		0.7 [0.4-1.5]		1.2 [0.6-2.4]	
G carriers	67 (0.73)	105 (0.77)	78 (0.68)	1.2 [0.6-2.2]	0.59	0.8 [0.4-1.5]	0.54	0.7 [0.4-1.2]	0.20	1.0 [0.6-1.7]	0.99

a odds ratio (OR) values shown are corrected for gender and ethnicity; results are shown as n (frequency); CI: confidence interval; total genotype counts can vary due to different genotypic call rates.

*Meta-analysis on the −308G>A TNF gene and association with Chagas' heart disease* - After a literature search, we observed that studies were not converging and that results for the association of CD and cytokine polymorphisms were controversial. We attempted to decrease ambiguity and searched for studies to conduct a meta-analysis. Selection of published articles for the meta-analysis resulted in 13 references that evaluated the same markers tested in the present study [Supplementary data (Fig. 2)]. Unfortunately, the reduced quantity of studies only allowed a meta-analysis of *TNF* rs1800629 −308G>A (N = 5 studies), comparing asymptomatic (A) *vs.* severe CCC (C) patients in Brazilian, Colombian, Mexican, and Peruvian populations (Beraún et al. 1998, Rodríguez-Pérez et al. 2005, Drigo et al. 2007, Pissetti et al. 2011, Criado et al. 2012), as summarised in Supplementary data (Table IV). After testing the inclusion/exclusion criteria, it was verified that the asymptomatic group in the study by Rodríguez-Pérez et al. (2005) did not follow HWE, and therefore was excluded from further analysis. The forest plots for allele, carrier, and genotype comparisons (Fig. 2) showed that the OR values and confidence intervals were not statistically significant. Consensus values for the published studies for the random model effects analysis are detailed in Table VI and depict a risk association for A allele (OR = 1.7, p*-*value = 0.02) and carriers of the minor allele A (OR = 2.1, p*-*value = 0.02). However, when the current study was included in the meta-analysis (group A, asymptomatic, 110 patients; group C, severe CCC, 133 patients), reaching a total of 534 CCC and 472 asymptomatic patients, the association described above did not retain significance for A allele or A carriers (p-values = 0.14 and 0.15, respectively), as shown in Table VI and Fig. 2.

**TABLE VI:** Summary of meta-analysis of published studies for *TNF* −308 G > A (rs1800629) and chagasic cardiomyopathy^a^

	Meta-analysis of published studies				Meta-analysis of published studies and present data			
*TNF* −308 G > A rs1800629	CCC N = 424	ASY N = 339	OR^b^ [95% CI]	p*-*value	CCC N = 534	ASY N = 472	OR^b^ [95% CI]	p-value
Allele A	124	60	1.7 [1.1 - 2.6]	0.02	147	83	1.4 [0.9 - 2.3]	0.14
A carriers	117	56	2.1 [1.1 - 3.1]	0.02	139	78	1.5 [0.9 - 2.6]	0.15
AA Genotype	7	4	1.3 [0.4 - 4.5]	0.70	8	5	1.2 [0.4 - 3.7]	0.77

a results are shown for random-effects model;

b odds ratio (OR);

CCC: chronic chagasic cardiomyopathy; ASY: asymptomatic group; CI: confidence interval; the study by Rodríguez-Pérez et al. (2005) was excluded due to deviations from HWE in the control group.

## DISCUSSION

Out of the 13 cytokine-related polymorphisms that were studied for susceptibility to or progression of CCC, only *BAT1* −22 C>G (rs3853601) and *IFNG* +874 T>A (rs2430561) remained borderline significant for protection in the logistic regression analysis with adjustment for non-genetic covariates. Neither *TGFB* rs8179181, rs8105161, rs1800469), *IL10* (rs1800890/rs1800896/rs1800871), nor *TNF-BAT1-LTA* (rs1800629/rs3853601/rs909253/rs2239704) analysed haplotypes displayed significant associations with Chagas' heart disease outcome.

The genetic variants in *TGFB1* −509 C>T (rs1800469) and +10 T>C (rs1800470) have been associated with susceptibility to *T. cruzi* infection, but not to CCC, in Colombians and Peruvians (Calzada et al. 2009). However, we were not able to corroborate these results in the *TGFB* cluster (+20743 C>T, +19318 A>G, −509 C>T), when searching for an association with risk of or progression to severe CCC. Regarding the regulatory cytokine IL-10, a study in Brazilians showed that *IL-10* −1082 A allele was correlated with low IL-10 expression and CCC susceptibility (Costa et al. 2009). Conversely, as previously shown in a Colombian population (Flórez et al. 2011), the present study on CD patients born and resident at the northeast of Brazil showed no association of alleles and genotypes in the *IL-10* cluster with clinical outcome of CD.

In Colombians, the *IFNG* +874T allele has been associated with risk of developing CD *per se* but not CCC (Torres et al. 2010). Interestingly, in the present study, we observed an association with protection of developing the cardiac form of disease in the genotype comparison (A *vs.* B1, p = 0.03; A *vs.* B1+C, p = 0.02), and a borderline result for this protective effect for carriers of the A allele (A *vs.* B1 and A *vs.* B1+C, p = 0.06). These discrepant findings may be the result of a difference in genotypic distributions between Brazilian and Colombian populations. In our setting, the Brazilian CD patients present AA as the minor genotype (Freq = 0.16), whereas, in patients from Colombia, this is reversed, with TT as the minor genotype (Freq = 0.11). Thus, these data indicate that if we maintained the same baseline for comparison (e.g., TT) then both studies would point to an association with protection, therefore, suggesting that the A allele associated with lower IFNγ production could be acting as a beneficial factor in infection, and, in our case, be associated with progression to milder forms of Chagas' heart disease. Interestingly, low IFNγ serum levels were detected in CD patients with the indeterminate form and high IFNγ concentrations associated with Chagas' heart disease (Sousa et al. 2014). Further, IFNγ-inducible genes were upregulated in cardiac tissue from CCC patients (Cunha-Neto and Chevillard 2014); however, murine model studies suggest a higher level of complexity for the role of cytokines in chagasic cardiomyopathy. In acute experimental *T. cruzi*-infection, IFNγ is essential for parasite control (Aliberti et al. 2001). Nevertheless, in chronic infection, IFNγ may fuel *T. cruzi* invasion of host cells and parasite persistence in the central nervous tissue (Silva et al. 2015). Most intriguing, *T. cruzi*-specific CD8^+^IFNγ^+^ T cells, mostly accumulated in peripheral tissues (blood and spleen), play a protective role against tissue injury when able to migrate towards lesioned myocardium (Silverio et al. 2012).

In the present study, the *TNF* −308 (rs1800629) polymorphism showed no significant association with risk of or progression to severe Chagas' heart disease. Polymorphisms in the *TNF* gene were previously evaluated in populations from Peru, Mexico, Brazil, and Colombia, and results showed no consensus. In a study in Peruvian CD patients, markers at −308 (rs1800629), −244, and −238 (rs361525) showed no associations, when comparing asymptomatic *vs.* the CCC group. On the other hand, the *TNF* −308A allele showed a risk association, considering both CD disease *per se* and CCC susceptibility in Mexicans (Rodríguez-Pérez et al. 2005). In Brazilians, 42 CCC patients that were A allele carriers had a reduced mean survival time compared with non-carriers (Drigo et al. 2005). Unfortunately, the same group was not able to replicate this association in a later study (Drigo et al. 2007). Another study suggested a susceptibility association between CCC and *TNF* −238 SNP, but not *TNF* −308 (Pissetti et al. 2011). A more recent study in Colombians also included polymorphism *TNF* −1031 C>T (rs1799964) in the equation, showing that C allele carriers as well as −308A carriers displayed a significant association with CCC (Criado et al. 2012). TNF signals via TNF receptor 1 (TNFR1/p55) and TNFR2 (p75). TNFR1, but not TNFR2, has been shown to control acute *T. cruzi* infection (Aliberti et al. 2001). Recently, TNF/TNFR1 signalling has been associated with increased TNF production and cardiac tissue damage (Vilar-Pereira et al. 2016). A previous report indicated that the *TNFR2* +676 G>T SNP, which was associated with lower TNFR2 expression, was monomorphic in the Colombian population (Criado et al. 2012). In our study neither *TNFR1* +36 A>G (rs767455) nor *TNFR2* +1466 A>G (rs1061624) showed a significant association with Chagas' heart disease outcome.

In addition to *TNF*, the chromosome 6p21 region encompasses other genes such as *LTA* and *BAT1* that have been evaluated in CD. LTA has a pro-inflammatory role and shares the same receptors as TNF (TNFR1 and TNFR2). *BAT1* −22 C>G (rs3853601) and −348 C>T variants, which alter the transcriptional activity of the gene itself, have been linked to CCC development (Ramasawmy et al. 2006). In CD, the *LTA* +252G (rs909253) and +80C (rs2239704) alleles independently or as a haplotype combination were associated with CCC susceptibility in a population composed of individuals from different geographical regions in Brazil (Ramasawmy et al. 2007). Moreover, in another Brazilian study, patients from the state of Minas Gerais showed a 2.8 times higher risk of developing CCC in *LTA* +252GG genotype carriers, and the G allele was associated with higher LTA production (Pissetti et al. 2013). However, in the present study, focused on a population born and residing in the state of Pernambuco in north-eastern Brazil, we were not able to corroborate these findings.

The correlation between higher TNF levels and worsening of CCC, stablished by a reduction in LVEF, has been described (Sousa et al. 2014). In our study, although TNF serum levels were increased in cardiopathic B1 and C patients compared with asymptomatic A patients, there was no correlation between TNF levels and decrease of LVEF. Functional characterisation of cytokine production and its association with disease phenotypes or with the polymorphism genotypes represents a challenge in CD. As previously stated, the complex structure of 6p21 could suggest that polymorphic sites other than −308 (rs1800629) affect TNF levels (Ramasawmy et al. 2007). A meta-analysis of functional studies focusing on the influence of −308 on TNF levels showed no effect at the mRNA level (Mekinian et al. 2011). Indeed, our study reveals no association between TNF expression and the *TNF* −308 (rs1800629) genotype. Altogether, these findings suggest that TNF may be merely a component of a complex inflammatory profile associated with cardiomyopathy in CD, rather than a single determinant factor.

Although it is tempting to suggest the possible influence of *BAT1* −22 (rs3853601) and *IFNG* +874 (rs2430561) polymorphisms in Chagas' heart disease outcomes in the present work, the interactions with other cytokines and their genetic variants could also participate in determining these outcomes. In fact, *MIF*, *IL1B*, *IL1RN*, *IL4*, and *IL12* SNPs also showed an association with disease susceptibility and CCC progression in Latin American CD populations (Ayo et al. 2013). Further, the apparent association with high/low producing alleles is not straightforward when translated to functional cytokine levels, and this should be studied further. Additionally, one should keep in mind that in CD, cytokine production may be a consequence of more complex parasite/host interplay. In CD, the fine balance that controls the pathogen and sustains an efficient immunologic response may rely on host genetic and non-genetic factors. The complex interaction with the parasite has proven to be relevant, as different *T. cruzi* strains have been associated with differential chronic outcomes (Cura et al. 2012, Rassi Jr et al. 2017).

Because of such results, we performed a literature search of the SNPs evaluated in our study. The number of studies on CD was low; therefore, we could only carry out a meta-analysis of TNF studies with the objective of attaining a consensus in OR estimates. In the Mexican study by Rodríguez-Pérez et al. (2005), the control group (asymptomatic) did not follow HWE assumptions and therefore was excluded from analysis. Our summary results initially showed a significant risk association for −308 (rs1800629) A allele and A carriers. After inclusion of our study, although the number of individuals was increased to 534 for CCC and 472 for asymptomatic patients, there were no consensus in the pooled OR estimates, and the significance from previous comparisons was lost. This could be because of the different clinical classifications that each study considered in determining patients asymptomatic and having CCC. In addition, the number of published articles on *TNF* −308 (rs1800629) gene polymorphisms in CD is low compared to studies on other chronic infectious diseases such as tuberculosis, for which more than 15 studies were available (Wang et al. 2012). Therefore, these data reinforce the need of more independent association studies in CD that evaluate the clinical associations with this and other polymorphisms, as well as the importance of standardising clinical outcomes for CD patients.

One limitation of most of the studies on gene polymorphisms in CD is sample size, and we also consider this feature a limitation in our study, with borderline association results impacted by adjustment for multiple testing. However, we show that the sample evaluated in the present work represents a population that is geographically distinct from those in previously published literature on CD and genetic association studies. In addition, a relevant point in our study is the strict clinical classification of CD patients. For decades, the criteria defining the different stratification and prognostic tests for the chronic phase remained highly debatable. In 2009, a systematic review highlighted three principal prognostic estimators: functional group according to the New York Heart Association, cardiac enlargement (cardiomegaly), and a reduction in the left ventricle ejection fraction (% LVEF) (Rassi Jr and Rassi 2010). To normalise the stratification in clinical groups of CCC patients, after consensus, the I Latin American Guideline for the Diagnosis and Treatment of Chagas' Heart Disease was published (Andrade et al. 2011). These standardised parameters and functional groups were adopted in the present study. Therefore, differences in clinical characterisations could have reduced the homogeneity of the groups under comparison in the different studies included in the meta-analysis.

Regarding the *TNF* −308 (rs1800629) polymorphism, and considering the described results, it seems that the region represents complexity that it is not well resolved in recent studies. The *TNF* gene is contained in the telomeric portion adjacent to HLA III. Many SNPs in this region have been shown to be in linkage disequilibrium with HLA II. In fact, HLA-DRB1 alleles were associated with CCC susceptibility in several populations (Williams-Blangero et al. 2011, Ayo et al. 2013). Overall, it seems that this chromosomic region requires a higher density of markers and testing with larger study populations.

Potential polymorphisms that could serve as prognostic markers to indicate groups prone to developing severe cardiac manifestations of CD would be of interest. This is especially true for this disease, considering the long period of time for presentation of clinical symptoms of progression. The genetic, functional, and systematic review strategies explored in our study pointed to some borderline genetic associations. Overall, our study indicates the difficulty of characterising the influence of genetic variants localised in immune response genes relevant to CD outcome. The absence of a strong genetic biomarker may indicate that the consequences of an immune response, including parasite control and the inflammatory profile, for CD patients are not genetically predetermined. Therefore, this lack of evidence may stimulate further studies and support the search for physiopathological factors resulting from parasite/host interactions that contribute to Chagas' heart disease outcome and severity. This approach may lead to therapeutic strategies to modify this interplay, ameliorating the prognosis and improving the quality of life for CD patients.
