# Supplementary material for: Single nucleotide polymorphisms of cytokine-related genes and association with clinical outcome in a Chagas disease case-control study from Brazil
Source: Mem Inst Oswaldo Cruz. 2018 May 14;113(6):e170489. doi: 10.1590/0074-02760170489 (PMC5961924; doi:10.1590/0074-02760170489)
Supplement: Supplementary file 1 [file 0074-0276-mioc-113-6-e170489-Suppl01.pdf]

Mem Inst Oswaldo Cruz, Rio de Janeiro, Vol. 113(6): e1704891, 2018

TABLE I

Details of the TaqMan assays* used for single nucleotide polymorphisms (SNPs) genotyping

Gene	Location[b]	SNP	A > a	dbSNP	TaqMan assay ID
TGFB1	chr 19: 41332301	+20743	C > T	rs8179181	C___7818404_20
TGFB1	chr 19: 46531471	+19318	A > G	rs8105161	AH21DCB**
TGFB1	chr 19: 41354391	-509	C > T	rs1800469	C___8708473_10
IL10	chr 1: 206776020	-3575	T > A	rs1800890	C___8828790_10
IL10	chr 1: 206773289	-819	C > T	rs1800871	C___1747362_10
IL10	chr 1: 206773552	-1082	G > A	rs1800896	C___1747360_10
IFNG	chr 12: 68158742	+874	T > A	rs2430561	AHHS6SK**
TNF	chr 6: 31575254	-308	G > A	rs1800629	C___7514879_10
BAT1	chr 6: 31531826	-22	C > G	rs3853601	C___2215712_10
LTA	chr 6: 31572536	+252	A > G	rs909253	C___2451911_10
LTA	chr 6: 31572364	+80	A > C	rs2239704	C___2451912_1_
TNFR1	chr 12: 6341779	+36	A > G	rs767455	C___2298465_20
TNFR2	chr 1: 12207208	+1466	A > G	rs1061624	C___8861229_10

*: ThermoFisher Scientific, USA; **: refers to custom designed assays; *b*: location is based on GRCh38 assembly build; A: ancestral allele; a: minor allele.

TABLE II

Haplotype analysis of single nucleotide polymorphisms (SNPs) located in the *IL10* cluster. Haplotype frequencies are shown by group

rs1800896/rs1800890/rs1800871				A versus B1		A versus C		B1 versus C	
	Stage A N = 110	Stage B1 N = 163	Stage C N = 133	OR[a] [95% CI]	p-value	OR[a] [95% CI]	p-value	OR[a] [95% CI]	p-value
G/A/C	0.37	0.31	0.28			Reference			
A/A/C	0.08	0.10	0.09	1.6 [0.7 - 3.3]	0.22	1.2 [0.5 - 2.7]	0.65	0.8 [0.4 - 1.6]	0.55
A/T/C	0.22	0.19	0.21	1.2 [0.7 - 2.1]	0.49	1.3 [0.7 - 2.2]	0.40	1.0 [0.6 - 1.7]	0.93
G/A/T	0.30	0.35	0.37	1.1 [0.6 - 1.9]	0.74	1.4 [0.8 - 2.4]	0.30	1.1 [0.7 - 1.8]	0.56
G/T/C	0.03	0.04	0.03	1.2 [0.4 - 3.6]	0.70	1.3 [0.3 - 4.1]	0.80	0.9 [0.3 - 2.4]	0.80

a: odds ratio (OR) values shown are corrected for gender and ethnicity; CI: confidence interval.

TABLE III

Haplotype analysis of single nucleotide polymorphisms (SNPs) located in the *TNF-BAT-LTA* cluster. Haplotype frequencies are shown by group

rs1800629/rs3853601/rs909253/rs2239704				A versus B1		A versus C		B1 versus C	
	Stage A N = 110	Stage B1 N = 163	Stage C N = 133	OR[a] [95% CI]	p-value	OR[a] [95% CI]	p-value	OR[a] [95% CI]	p-value
G/C/A/C	0.30	0.31	0.32			Reference			
A/C/G/C	0.09	0.08	0.07	0.7 [0.3 - 1.5]	0.34	0.6 [0.3 - 1.5]	0.30	0.9 [0.4 - 1.9]	0.83
G/C/A/A	0.35	0.33	0.32	0.8 [0.5 - 1.3]	0.34	0.8 [0.5 - 1.3]	0.37	1.1 [0.7 - 1.7]	0.70
G/C/G/C	0.15	0.14	0.15	0.7 [0.4 - 1.3]	0.22	0.8 [0.4 - 1.5]	0.49	1.2 [0.7 - 2.2]	0.50
G/G/G/C	0.09	0.13	0.09	1.2 [0.6 - 2.4]	0.55	0.6 [0.3 - 1.3]	0.24	0.6 [0.3 - 1.1]	0.10

a: odds ratio (OR) values shown are corrected for gender and ethnicity; CI: confidence interval.

TABLE IV

Summary of the data obtained from the selected papers for meta-analysis

	Chronic chagasic cardiomyopathy						Asymptomatic				
Author, year	Population	GG	GA	AA	Total	HWE	GG	GA	AA	Total	HWE
Beraún et al. (1998)	Peruvian	28 (85%)	5 (15%)	0 (0 %)	33	Yes	48 (92%)	4 (8%)	0 (0%)	52	Yes
Rodríguez-Pérez et al. (2005)	Mexican	16 (60%)	9 (33%)	2 (7%)	27	Yes	27 (100%)	0 (0 %)	0 (0 %)	27	No
Drigo et al. (2007)	Brazilian	131 (79%)	33 (20%)	2 (1%)	166	Yes	65 (81%)	14 (18%)	1 (1%)	80	Yes
Pissetti et al. (2011)	Brazilian	41 (62%)	21 (32%)	4 (6%)	66	Yes	37 (70%)	14 (26%)	2 (4%)	53	Yes
Criado et al. (2012)	Colombian	107 (67%)	51 (32%)	1 (1%)	159	Yes	133 (86%)	20 (13%)	1 (1%)	154	Yes

Genotype counts are shown as N (frequency); the study by Rodríguez-Pérez et al. (2005) was excluded due to deviations from Hardy-Weinberg equilibrium (HWE) in the control group.

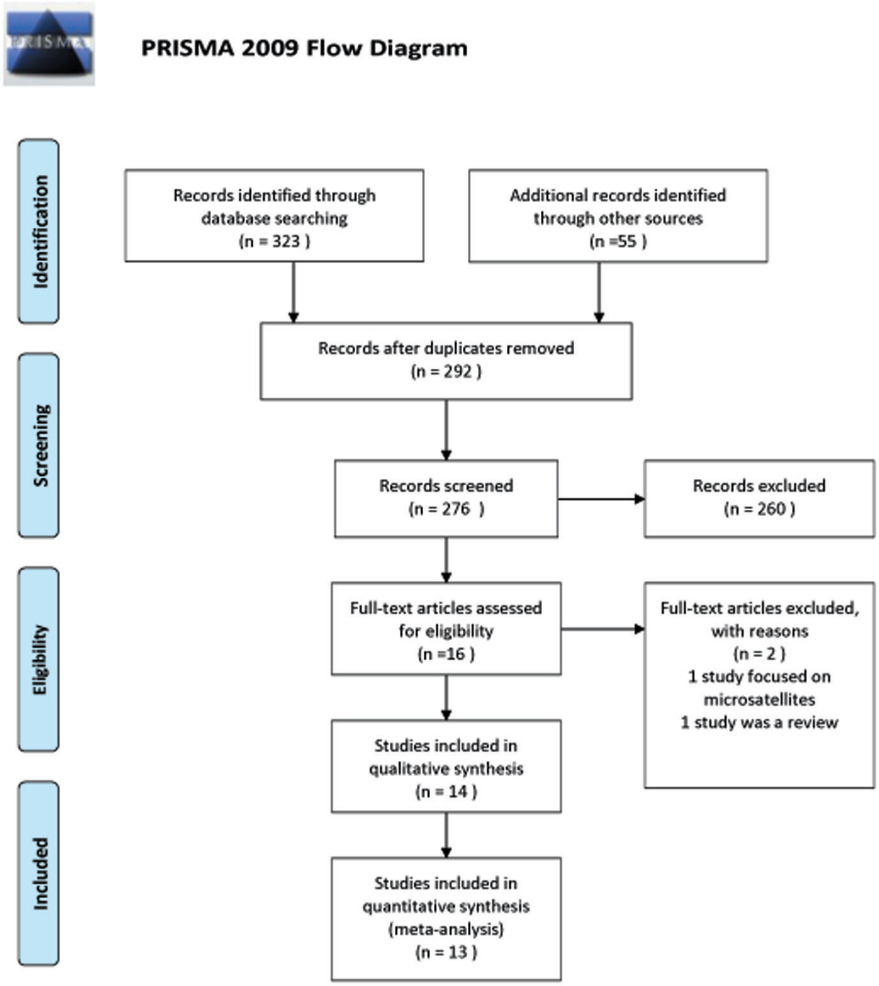

Fig. 1: PRISMA flow diagram. Source: Moher et al. (2009).

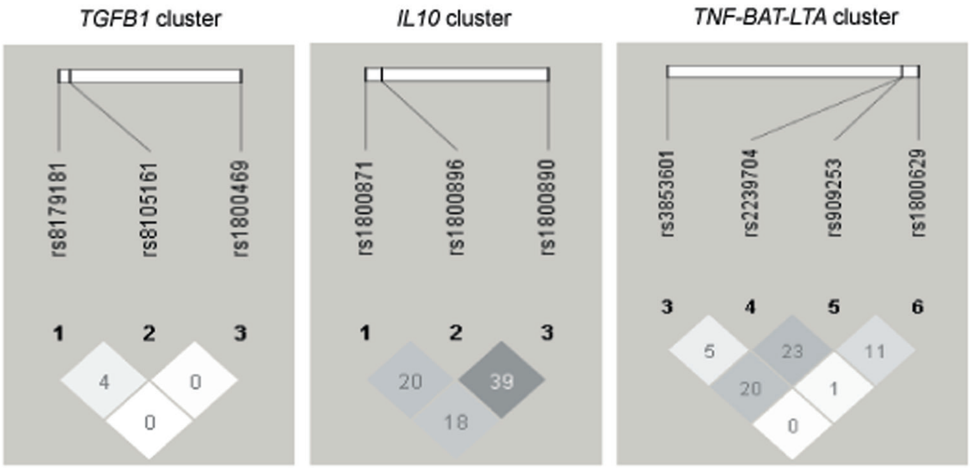

Fig. 2: linkage disequilibrium analysis for the tested polymorphisms and evaluating the Chagas disease patients recruited for the present study. Left: *TGFB* cluster; middle: *IL10* cluster; right: *TNF-BAT-LTA* cluster. Values shown in each box and the intensity of shading are proportional to r^2.
